# Whole-Genome Sequences of 24 *Brucella* Strains

**DOI:** 10.1128/genomeA.00915-14

**Published:** 2014-09-18

**Authors:** T. D. Minogue, H. A. Daligault, K. W. Davenport, K. A. Bishop-Lilly, S. M. Broomall, D. C. Bruce, P. S. Chain, O. Chertkov, S. R. Coyne, K. G. Frey, H. S. Gibbons, J. Jaissle, G. I. Koroleva, J. T. Ladner, C.-C. Lo, G. F. Palacios, C. L. Redden, C. N. Rosenzweig, M. B. Scholz, Y. Xu, S. L. Johnson

**Affiliations:** a Diagnostic Systems Division, U.S. Army Medical Research Institute of Infectious Diseases, Fort Detrick, Maryland, USA; b Los Alamos National Laboratory, Los Alamos, New Mexico, USA; c Naval Medical Research Center, NMRC-Frederick, Fort Detrick, Maryland, USA; d Henry M. Jackson Foundation, Bethesda, Maryland, USA; e U.S. Army Edgewood Chemical Biological Center, Aberdeen Proving Grounds, Maryland, USA; f Center for Genome Sciences, U.S. Army Medical Research Institute of Infectious Diseases, Fort Detrick, Maryland, USA

## Abstract

*Brucella* species are intracellular zoonotic pathogens which cause, among other pathologies, increased rates of abortion in ruminants. Human infections are generally associated with exposure to contaminated and unpasteurized dairy products; however Brucellae have been developed as bioweapons. Here we present 17 complete and 7 scaffolded genome assemblies of *Brucella* strains.

## GENOME ANNOUNCEMENT

*Brucella* are Gram-negative facultative intracellular pathogens with reduced genomes, as typical for intracellular *Alphaproteobacteria* (1, 2). Globally, *Brucella* spp. have a substantial impact on rural areas of the world, where surveillance and vaccination programs are lacking (3, 4). *Brucella* spp. express a surface lipopolysaccharide that contributes to pathogenicity. The bacteria reside within white blood cells (primarily macrophages) without disrupting the cell function and cycle (5, 6), and there is overall a reduced immune response as compared to most other Gram-negative bacteria. They are largely host-specific zoonotic pathogens, and humans may be infected by less than 100 cells (6–8). Disease in animals (ruminants) often causes abortion and/or sterility, but disease in humans is characterized by undulant fever, at times progressing into severe and/or incapacitating complications (9, 10). Human infections are most commonly attributed to unpasteurized dairy consumption, but brucellosis can also be transmitted through aerosols (6, 11, 12). Due to its ease of airborne transmission and chronic, difficult-to-treat pathology, *Brucella* spp. have been developed as bioweapons and are listed as CDC Category B pathogens (6, 13).

High-quality genomic DNA was extracted from purified isolates of each strain using QIAgen Genome Tip-500 at the U.S. Army Medical Research Institute of Infectious Diseases, Diagnostic Systems Division (USARMIID-DSD). Specifically, 100-mL bacterial cultures were grown to the stationary phase and nucleic acid was extracted per the manufacturer’s recommendations. For BSL3 *Brucella*, all extracted material was checked for sterility. If sterility was not achieved, then the nucleic acid was passed through a 0.45-µM filter and rechecked for viable organisms before removal from the BSL3 suite. Sequence data for each draft genome was generated using a combination of Illumina and 454 technologies (14, 15). For each genome, we constructed and sequenced an Illumina “standard” library of 100-bp reads at high coverage and a separate long-insert paired-end library (Roche 454 Titanium or Illumina platform). The two datasets were assembled together in Newbler (Roche) and the consensus sequences computationally shredded into 2-Kbp overlapping fake reads (shreds). The raw reads were also assembled in Velvet and those consensus sequences were computationally shredded into 1.5-Kbp overlapping shreds (16). Draft data from all platforms were then assembled together with ALLPATHS and the consensus sequences were computationally shredded into 10-Kbp overlapping shreds (17). We then integrated the Newbler consensus shreds, Velvet consensus shreds, Allpaths consensus shreds, and a subset of the long-insert read-pairs using parallel Phrap (High Performance Software, LLC). Possible misassemblies were corrected and some gap closure was accomplished with manual editing in Consed (18–20). Of the 24 genomes, 7 are scaffolded draft assemblies and 17 are closed “finished” genomes.

Automatic annotation for each genome utilized an Ergatis-based workflow at LANL with minor manual curation. Each genome is available in NCBI (accession numbers listed in Table 1) and the raw data can be provided upon request. In-depth comparative analyses of these genomes are currently under way and will be published in an upcoming manuscript.

**TABLE 1:** Listing of strains, accession numbers, and basic annotation statistics for each genome sequenced

Strain	Accession no.^*a*^	Size (bp)	%GC	Draft coverage	CDS	tRNA	rRNA
*Brucella abortus*							
870 BEV	CP007700 Chr I	2,123,615 Chr I	57.2	474	3,124	55	9
	CP007701 Chr II	1,161,664 Chr II					
870 BFX	CP007709 Chr I	2,124,096 Chr I	57.2	150	3,127	55	9
	CP007710 Chr II	1,157,058 Chr II					
2308	JMRZ00000000	3,269,686 total	57.2	793	3,124	52	5
	WGS (12)						
BEU	JMSA00000000	3,321,680 total	57.2	167	3,226	55	9
	WGS (5)						
BDW	CP007681 Chr I	2,128,683 Chr I	57.2	161	3,141	55	9
	CP007680 Chr II	1,160,817 Chr II					
292 DPG	JMSB00000000	3,262,739 total	57.2	272	3,135	49	3
	WGS (43)						
63 75	CP007663 Chr I	2,124,677 Chr I	57.3	176	3,112	55	9
	CP007662 Chr II	1,155,633 Chr II					
86/8/59	CP007765 Chr I	2,123,991 Chr I	57.2	327	3,434	55	9
	CP007764 Chr II	1,162,137 Chr II					
B3196	CP007707 Chr I	2,123,890 Chr I	57.2	342	3,113	55	9
	CP007708 Chr II	1,155,864 Chr II					
Tulya BER	CP007682 Chr I	2,125,180 Chr I	57.2	216	2,121	54	9
	CP007683 Chr II	1,163,338 Chr II					
Tulya BFY	CP007738 Chr I	2,124,832 Chr I	57.2	300	3,120	54	9
	CP007737 Chr II	1,163,326 Chr II					
C68	CP007705 Chr I	2,124,100 Chr I	57.2	119	3,111	55	9
	CP007706 Chr II	1,155,846 Chr II					
*Brucella canis* RM6/66	CP007758 Chr I	2,105,950 Chr I	57.2	320	3,108	55	9
	CP007759 Chr II	1,206,801 Chr II					
*Brucella melitensis*							
16 M	CP007762 Chr I	2,116,984 Chr I	57.2	762	3,140	54	9
	CP007763 Chr II	1,177,791 Chr II					
63/9	CP007789 Chr I	2,127,512 Chr I	57.2	152	3,156	55	9
	CP007788 Chr II	1,185,446 Chr II					
Ether	CP007760 Chr I	2,122,766 Chr I	57.2	321	3,156	55	9
	CP007761 Chr II	1,187,961 Chr II					
*Brucella neotomae* 5K33	JMSC00000000	3,328,864 total	57.2	321	3,199	55	11
	WGS (7)						
*Brucella pinnipedialis* 6/566	CP007743 Chr I	2,139,033 Chr I	57.3	217	3,137	55	9
	CP007742 Chr II	1,191,996 Chr II					
*Brucella suis*							
686	CP007719 Chr I	2,107,052 Chr I	57.2	304	3,091	55	9
	CP007718 Chr II	1,190,208 Chr II					
1330	JMUC00000000	3,294,601 total	57.3	319	3,124	51	3
	WGS (16)						
513UK	CP007717 Chr I	2,131,717 Chr I	57.3	300	3,091	55	9
	CP007716 Chr II	1,187,980 Chr II					
40 BSP	CP008757 Chr I	1,902,870 Chr I	57.2	790	3,101	55	9
	CP008756 Chr II	1,410,995 Chr II					
BSQ (40)	JMUD00000000	3,308,964 total	57.3	399	3,099	55	9
	WGS (5)						
Thompsen	JMUE00000000	3,316,531 total	57.2	314	3,152	52	6
	WGS (10)						

a Chr I, chromosome I; Chr II, chromosome II.

### Nucleotide sequence accession numbers.

Genome accession numbers to public databases are listed in Table 1.
